# Ischemic postconditioning ameliorates diabetic cerebral ischemia via activating the brain-derived neurotrophic factor–tropomyosin receptor kinase B–hypoxia-inducible factor 1α–Bcl-2/adenovirus E1B 19-kDa-interacting protein 3 pathway to induce microglial mitophagy and suppress A1 astrocyte-mediated neuroinflammation

**DOI:** 10.3389/fendo.2025.1620004

**Published:** 2025-09-22

**Authors:** Ling Zhao, Yuanyuan Han, Ying Wang, Tingyu Ke, Shiying Huang

**Affiliations:** ^1^ Department of Endocrinology, The Second Affiliated Hospital of Kunming Medical University, Kunming, Yunnan, China; ^2^ Institute of Medical Biology, Chinese Academy of Medical Science and Peking Union Medical College, Kunming, Yunnan, China; ^3^ Department of Endocrinology, The First Affiliated Hospital of Kunming Medical University, Kunming, Yunnan, China

**Keywords:** ischemic postconditioning, diabetes, cerebral ischemia, BDNF-TrkB-HIF-1α-BNIP3, neuroprotective

## Abstract

**Aim:**

Diabetes mellitus exacerbates cerebral ischemic injury. However, effective interventions remain limited. Ischemic postconditioning (IPOC) is a potential neuroprotective strategy; however, its efficacy and mechanisms in diabetes remain poorly understood. This study aimed to explore the therapeutic effects and underlying mechanisms of IPOC in diabetes complicated by cerebral ischemia.

**Methods:**

Tree shrews with diabetes complicated by cerebral ischemia were used as the study subjects and were subjected to a standardized IPOC intervention protocol.

**Results:**

The results showed that compared with the control group, the cerebral infarction volume of tree shrews in the cerebral ischemia (IS) group and the diabetes complicated with cerebral ischemia (DMIS) group was significantly higher, the neurons were severely damaged, A1 astrocytes were activated, the levels of inflammatory factors interleukin (IL)-1β and IL-6 increased, and mitochondrial autophagy was inhibited. In contrast, in the DMIS + IPOC group, cerebral infarction volume was significantly reduced, neuronal damage was improved, activation of A1 astrocytes and release of inflammatory factors were inhibited, and mitochondrial autophagy was increased. Mechanistically, IPOC activated the brain-derived neurotrophic factor (BDNF)/tropomyosin receptor kinase B (TrkB) pathway and upregulated hypoxia-inducible factor 1α (HIF-1α) expression, which further promoted the expression of Bcl-2/adenovirus E1B 19-kDa-interacting protein 3 (BNIP3).

**Conclusion:**

IPOC coordinates microglial mitochondrial autophagy and astrocyte inflammatory regulation through the BDNF–TrkB–HIF–1α–BNIP3 signaling cascade, providing a new target for precise intervention in diabetes combined with cerebral ischemia.

## Introduction

Diabetes mellitus, a major challenge in global public health, has shown a continuous upward trend in its prevalence (1). Among its numerous complications, diabetes-related cerebral ischemia has received considerable attention among the numerous complications of diabetes owing to its high disability and mortality rates (2, 3). Studies on pathological mechanisms have shown that during cerebral ischemia, a cascade effect is formed by energy metabolism disorders, excitotoxicity of excitatory amino acids, oxidative stress, and inflammatory responses (4–7). The inflammatory mechanism exacerbates nerve damage through pathways such as mediating the formation of cerebral edema, altering the permeability of the blood–brain barrier, promoting the infiltration of inflammatory cells, and causing abnormal expression of adhesion molecules (8, 9). Notably, metabolic disorders in diabetes can significantly magnify the aforementioned pathological processes, resulting in a higher incidence of cerebral ischemia in patients with diabetes than in individuals without diabetes (10, 11). Clinically, patients with diabetes and cerebral ischemia are characterized by extensive infarct lesions, severe neurological deficits, and poor prognosis (12).

Current clinical intervention strategies mainly focus on revascularization in the acute phase, including vascular recanalization techniques such as intravenous thrombolysis and endovascular mechanical thrombectomy (13). However, their effectiveness is limited by the strict time window (< 4.5 h) and secondary injuries, such as oxidative burst and calcium overload, which may be induced after reperfusion (14). Although basic treatments, such as blood glucose regulation, can partially improve metabolic disorders, it remains difficult to reverse the unique “metabolism–inflammation–vascular” interactive injury mechanism of cerebral ischemia in the context of diabetes. Therefore, the development of innovative treatment strategies with multitarget regulatory effects has become a research focus.

Ischemic postconditioning (IPOC), an intervention method that implements a programmed ischemia–reperfusion cycle at the initial stage of reperfusion, has a significant protective effect on important organs, such as the heart and brain, by regulating the inflammatory response, maintaining autophagic homeostasis, and activating survival signaling pathways, such as phosphoinositide 3-kinase (PI3K)/protein kinase B (Akt) (15, 16). IPOC may regulate multiple cellular and molecular pathways, including anti-inflammatory responses, autophagy regulation, and activation of survival signaling cascades. However, chronic inflammation, abnormal mitochondrial function, and impaired endogenous protective mechanisms caused by diabetes may affect the neuroprotective efficacy of IPOC, and it remains unclear whether IPOC exerts similar beneficial effects (17, 18). Previous studies have largely overlooked the impact of diabetes-induced glial activation and mitochondrial impairment in modulating IPOC outcomes, leaving a critical gap in our understanding of its potential effects under metabolic stress conditions. To address this gap, this study used a tree shrew model of diabetes combined with cerebral ischemia to explore the effects and mechanisms of IPOC on diabetes complicated by cerebral ischemia, with the hope of providing new insights and potential therapeutic targets for this challenging disease.

## Materials and methods

### Animals and grouping

Healthy male tree shrews at the general level, aged 7–12 months with a body weight of 130 ± 30 g, were purchased from Yunnan Zeren Biotechnology Co., Ltd. The production license for the experimental animals was SCXK (Dian) 2024-0002. All animal procedures were approved by the Experimental Animal Welfare and Medical Ethics Committee of Kunming Medical University. The ethical review approval number was Kmmu2021099 and the license number was SYXK (Dian) K2023-0009. The animals were raised following the 3R principles and welfare ethics principles for experimental animals. The rearing conditions were as follows: humidity, 50%–70%; temperature, 20–25°C; and a 12-h light/12-h dark cycle. All animals had free access to food and water ad libitum.

Twenty-four tree shrews were randomly divided into four groups; a control group (control), cerebral ischemia group (IS), diabetes combined with cerebral ischemia group (DMIS), and diabetes combined with cerebral ischemia group with IPOC (DMIS + IPOC), with six tree shrews in each group. The experimental timeline for all groups is summarized in **Supplementary Figure S1**. Briefly, diabetes induction was initiated 3 weeks prior to stroke induction, and IPOC was administered immediately after the 4-h ischemia period. Tissue collection was performed at 72 h post-IPOC intervention. The right common carotid artery was isolated in the control group without clamping. In the IS group, a focal thrombotic cerebral ischemia tree shrew model was established using photochemical induction. In the DMIS group, a tree shrew diabetes model was successfully established using a high-fat diet combined with the streptozotocin (STZ) method, and a focal thrombotic cerebral ischemia model was constructed by photochemical induction. In the DMIS + IPOC group, the animals were anesthetized in the first 40 min after 4 h of ischemia and were fixed in the supine position. The right carotid sheath was separated from the anterior edge of the right sternocleidomastoid muscle, and the right common carotid artery was also separated. In the first 30 min after 4 h of ischemia, the common carotid artery was clamped with a non-invasive arterial clip at the upper edge of the thyroid cartilage for 5 min. Subsequently, the arterial clip was removed for 5 min and alternated for three cycles for a total duration of 30 min.

### Model establishment

After 1 week of adaptive feeding, the animals were fed a high-fat diet (69% basic feed supplemented with 1% cholesterol, 10% lard, 10% white sugar, and 10% fructose, mixed and steamed) for 1 week and then intraperitoneally injected with 2% STZ dissolved in 0.1% citrate-sodium citrate buffer at a dose of 100 mg/kg body weight. The control group was injected with an equal volume of citrate-sodium citrate buffer. Blood glucose levels in the tree shrews were measured daily after STZ injection. A blood glucose level ≥ 16.7 mmol/L indicated a successful model. For tree shrews that failed to be modeled, STZ was continuously injected, and blood glucose levels were detected. Four consecutive days after STZ injection, the blood glucose levels of all tree shrews increased. Detailed models for all animals in each group are provided in **Supplementary Figure S2**, confirming stable hyperglycemia in the DMIS and DMIS + IPOC groups throughout the experiment.

For induction of focal thrombotic cerebral ischemia, tree shrews were anesthetized with 3% isoflurane (induction) and 1.5–2% isoflurane (maintenance) in 100% oxygen (flow rate: 1 L/min) via a nose cone. Following confirmation of adequate anesthesia (loss of pedal reflex), the animals were placed in a stereotaxic frame (RWD Life Science, Shenzhen, China) to stabilize the head. A midline incision (~1 cm) was made on the right scalp, and the underlying periosteum was gently removed to expose the skull. A circular craniotomy (diameter: 0.5 cm) was performed using a dental drill (Fine Science Tools, Foster City, CA, USA) at coordinates 2.0 mm anterior to bregma, 3.0 mm lateral to the midline (19), avoiding damage to the dura mater. A sterile aluminum window (0.5 cm diameter) was affixed to the craniotomy site using cyanoacrylate glue to facilitate light transmission. Subsequently, rose bengal (Sigma-Aldrich, St. Louis, MO, USA; Cat. R2751) was dissolved in 0.9% saline at a concentration of 10 mg/mL and injected intravenously via the femoral vein at a dose of 1.33 mL/kg (equivalent to 13.3 mg/kg). After allowing 10 min for systemic circulation, the aluminum window was irradiated with a green laser (wavelength: 560 nm, intensity: 1.0 W/cm²) using an SQ-III cerebral thrombosis device (Huaibei Zhenghua Biological Instrument Co., Ltd., China). The laser was positioned perpendicular to the skull surface, and irradiation was maintained for 15 min. A feedback temperature controller (Physitemp, Clifton, NJ, USA) was used to keep the skull surface temperature at 36°C ± 1°C throughout irradiation to prevent thermal injury (20). The control group underwent identical procedures, including scalp incision, craniotomy, and rose bengal injection, but without laser irradiation.

### Cerebral infarct area

The 2,3,5-triphenyltetrazolium chloride (TTC) staining method was used. At 72 h after the IPOC intervention, the brain tissue of the tree shrew was collected, quick-frozen at –20°C for 30 min, and then cut into 4–6 slices with a thickness of approximately 2 mm along the coronal plane. The cut tissue slices were sequentially placed in a 2% TCC solution and stained at 37°C in the dark for 30 min. After the tissue turned dark red, a fixative was added for 24-h fixation. The tissue slices were removed, the fixative was blotted dry with filter paper and photographed, and the cerebral cortex and infarct volume were measured using NIH ImageJ software. The red part represents normal brain tissue, and the pale or gray part represents infarcted tissue and the penumbra. To correct for potential edema-induced volume changes, the infarct volume was calculated using the following formula: infarct volume percentage = (volume of the contralateral hemisphere–volume of non-infarcted tissue in the ipsilateral hemisphere)/volume of the contralateral hemisphere × 100%.

To ensure reproducibility, all image analyses were performed by two independent investigators who were blinded to the experimental groups. For each sample, infarct volume was measured in triplicate across consecutive tissue slices, and the mean value was used for statistical analysis. The intra-class correlation coefficient between the two investigators was >0.90, confirming high inter-observer consistency.

### Brain tissue pathology by hematoxylin–eosin staining

Brain tissue pathology was assessed using hematoxylin and eosin (H&E) staining. The brain tissue of the mice was fixed with 4% paraformaldehyde for > 24 h, and paraffin embedding and sectioning were performed. Subsequently, the paraffin sections were dewaxed in xylene I and II for 10 min each, hydrated in alcohol solutions with concentration gradients of 100%, 95%, 90%, 80%, and 70% for 5 min each, and soaked in distilled water for 3 min. Subsequently, the hematoxylin staining solution was added to the sections for 3–5 min, and the sections were rinsed with running tap water for approximately 3 min to make them turn blue (or placed in alkaline water), while ensuring that the water flow was not significantly strong. Subsequently, the sections were placed in 1% hydrochloric acid alcohol for differentiation for several seconds until the sections turned red and the color was relatively light. They were then placed in running tap water for blue return for several seconds and washed with water for 3 min. The sections were then counterstained with eosin staining solution for 3–5 min. After counterstaining, the sections were sequentially placed in absolute ethanol I (5 min), absolute ethanol II (5 min), absolute ethanol III (5 min), xylene I (5 min), and xylene II (5 min) for dehydration and clearing. Finally, the sections were sealed with neutral balsam, subjected to microscopic examination, and images were collected and analyzed.

### Immunohistochemical detection of specific markers of A1 astrocytes

For the immunohistochemical (IHC) detection of A1 astrocyte-specific markers, the procedure commenced with routine dewaxing of sections in water. Endogenous peroxidase was blocked by adding 3% hydrogen peroxide, which was placed in a wet box at room temperature in the dark for 15 min, followed by rinsing with phosphate-buffered saline (PBS) three times for 3 min each. Antigen retrieval was achieved by placing the slides in a preheated ethylenediaminetetraacetic acid (EDTA)/sodium citrate/EDTA-sodium citrate antigen retrieval solution for 10 min, after which they were allowed to cool to room temperature and rinsed thrice with PBS for 3 min each. Next, 5% goat serum + 0.3% Triton was added to completely cover the tissue, and the samples were incubated in a 37°C oven for 30 min or at room temperature for 2–3 h for blocking. After removing the blocking solution, the corresponding primary antibody was directly added to fully cover the tissue and incubated overnight at 4°C or for 2 h at 37°C. The wet box was then removed, and once it returned to room temperature, the sections were rinsed three times with PBS for 3 min each. Subsequently, horseradish peroxidase-conjugated secondary antibody was added to completely cover the tissue and incubated at room temperature for 20 min, followed by three 3-min PBS rinses. For chromogenic development, a freshly prepared 3,3’-diaminobenzidine chromogenic solution was added to cover the tissue, and the color was developed at room temperature for 3–5 min (depending on the color), with subsequent rinsing with PBS three times for 3 min each. Hematoxylin was used for counterstaining for 5 min (depending on the color), followed by rinsing with tap water. Differentiation was performed using the differentiation solution for several seconds, followed by rinsing with running tap water. The sections were then rinsed with running tap water for 3 min to obtain a blue return. Dehydration was performed by placing the sections in 75%, 85%, and 95% absolute ethanol for 1 min each, followed by clearing in xylene I and II for 1 min each. Finally, an appropriate amount of neutral balsam was added, and a coverslip was placed for the mounting.

### Mitochondrial autophagy in brain tissue in the ischemic area by transmission electron microscopy

For transmission electron microscopy (TEM) analysis of mitochondrial autophagy in ischemic brain tissue, the following steps were performed. First, tissue sampling and fixation were performed. The sampling site of the fresh tissue was determined to minimize mechanical damage such as pulling, contusion, and extrusion. Sampling was completed within 1–3 min, and the sampled tissue was 1 mm^3^ in size. A petri dish filled with an electron microscope fixative was prepared in advance. After excising the small tissue block, it was immediately placed in a Petri dish and cut into 1 mm^3^ pieces in the fixative using a scalpel. The cut tissue pieces were then transferred to an EP tube with fresh fixative for continued fixation at 4°C during storage and transportation. The tissue was then rinsed three times with 0.1 M PBS (pH 7.4) for 15 min each. Post-fixation was conducted using 1% osmium acid prepared in 0.1 M PB (pH 7.4) at room temperature in the dark for 2 h, followed by three 15-min rinses with 0.1 M PB (pH 7.4). At room temperature, the tissues were dehydrated using a series of alcohol solutions (30%, 50%, 70%, 80%, 95%, 100%, and 100%) for 20 min each and twice in 100% acetone for 15 min each. For infiltration and embedding, the tissue was first treated with a 1:1 mixture of acetone and 812 embedding agent at 37°C for 2–4 h, followed by a 1:2 mixture at 37°C overnight, and finally with pure 812 embedding agent at 37°C for 5–8 h. The pure embedding agent was poured into an embedding plate, and the sample was inserted and kept in a 37°C oven overnight. The embedding plate was then placed in a 60°C oven for 48-h for polymerization, and the resulting resin block was removed for use. Ultrathin sections (60–80 nm) were cut from the resin block using an ultramicrotome and were picked using a 150-mesh Fanghua membrane copper grid. The copper grids were stained with 2% uranyl acetate in a saturated alcohol solution in the dark for 8 min, washed thrice with 70% alcohol, and then thrice with ultrapure water. They were further stained with a 2.6% lead citrate solution while avoiding carbon dioxide for 8 min, followed by three washes with ultrapure water, and then slightly blotted dry with filter paper. Copper grid sections were placed in a grid box and dried overnight at room temperature. Finally, the samples were observed under a transmission electron microscope, and the images were collected and analyzed.

### Quantitative reverse transcription polymerase chain reaction

Reverse transcription polymerase chain reaction (RT-qPCR) was used to detect changes in the expression of target genes in brain tissue. First, 50 mg of fresh brain tissue was collected and 1 mL of VeZol Reagent was added. The tissue was homogenized using a tissue grinder until no evident tissue clumps were observed, and then left at room temperature for 5 min. Subsequently, 1/5 volume (200 µL) of chloroform was added, and the mixture was vigorously shaken for 15 s to form an emulsion. After incubation at room temperature for 5 min, the solution was centrifuged at 11,200 rpm (12,000 × g) at 4°C for 15 min. The upper aqueous phase (approximately 500 µL) was carefully aspirated into a new RNase-free centrifuge tube. An equal volume of isopropanol was then added. After mixing, the mixture was left at room temperature for 10 min and centrifuged under the same conditions for 10 min. The supernatants were then discarded. Subsequently, 1 mL of precooled 75% ethanol was added to resuspend the precipitate. After centrifugation at 11,200 rpm (12,000 × g) at 4°C for 5 min, the supernatant was discarded. The precipitate was air-dried at room temperature for 2–5 min and then dissolved in 30 μL RNase-free ddH_2_O. The total RNA concentration was measured using a microplate spectrophotometer. The volume of total RNA required was calculated based on the requirement of 1000 ng of total RNA to synthesize 20 µL of cDNA. The total RNA was aliquoted (10 μL per tube) and stored at –80°C. When the OD_260_/_280_ ratio was between 1.8 and 2.2, the RNA had good purity, and the sample was used.

Sixteen 200 μL centrifuge tubes were numbered. A mixture containing RNase-free ddH_2_O and 5 × gDNA wiper Mix, and the calculated amount of template RNA was prepared in an RNase-free centrifuge tube and incubated at 42°C for 2 min to remove the genomic DNA. Next, 4 × HiScript IV qRT SuperMix was added, and reverse transcription was performed using a real-time fluorescence quantitative PCR instrument (37°C for 15 min, 85°C for 5 s). The product can be immediately used for qPCR or stored at –20°C for up to 6 months. For long-term storage, it should be aliquoted and stored at –80°C, and repeated freeze-thaw cycles should be avoided.

ChamQ Universal SYBR qPCR Master Mix (2x), primers, cDNA, and ddH_2_O were added to a 96-well PCR plate according to the manufacturer’s instructions. The plate was placed in a real-time fluorescence quantitative PCR instrument, and the reaction conditions were established. After the reaction was complete, the Ct values were read, and the relative expression level of the gene was calculated using the 2^–△△Ct^ method. The primers used included upstream and downstream primer sequences of genes such as Trk-B, HIF-1a, BNIP3, BDNF, and actin (**Table 1**).

**Table 1:** The primer information.

Gene	Forward (5'-3')	Reverse (5'-3')
Trk-B	ACATTTCCGTCACCTCGACT	CCTGAGTTTCTGGACTGGGT
HIF-1a	GGTTGCATCTCCGTCTCCTA	ACACGTTAGGGCTTCTTGGA
BNIP3	CAAAGCGCACAGCTACTCTC	AGGTGCTAGTGGAGGTTGTC
BDNF	CAAAGCGCACAGCTACTCTC	AGGTGCTAGTGGAGGTTGTC
Actin	ATGGCTGACGACAGAGAGGAG	CTAGAAGCATTTGCGGAAGGT

### Western blotting

The samples were lysed on ice for 10 min and then centrifuged at 4°C and 14000 g for 15 min. After determining the protein concentration using a BCA protein quantification kit, 80 μL of protein was taken and mixed with 20 μL of 5 × protein loading buffer, boiled in a boiling water bath for 5 min, and then subjected to sodium dodecyl sulfate-polyacrylamide gel electrophoresis. After transfer, the polyvinylidene fluoride membrane was removed, and the cut strips wetted with 1×TBST were placed in a blocking solution (5% bovine serum albumin) and blocked on a shaker at room temperature for 0.5–1 h. The primary antibodies against LC3 II/I, Trkb, p-Trkb, BNIP3, p62, LC3 II/I, and BDNF, and the internal reference actin, were incubated at 4°C overnight, and the secondary antibody was incubated at room temperature for 60 min, developed, and photographed for preservation.

### Inflammatory factors in brain tissue detected by enzyme-linked immunosorbent assay

In total, 50 mg of brain tissue block was weighed, and 0.45 mL of precooled PBS was added, homogenized thoroughly with a grinder, and centrifuged at 10000 g at 4°C for 10 min. The supernatant was transferred to another EP tube, and 500 μL of chloroform was added, shaken well, freeze-thawed twice at –80°C, and centrifuged at 12000 × g at 4°C for 3 min. The supernatant was collected and stored at –80°C for later use. Repeated freeze–thaw cycles were avoided. The levels of inflammatory factors IL-1β and IL-6 in brain tissue were detected according to the instructions of the enzyme-linked immunosorbent assay (ELISA) kit.

### Statistical analyses

Data are expressed as the mean ± standard deviation. For data comparison between the two groups, an independent-sample t-test was used if the data conformed to a normal distribution; otherwise, a nonparametric test was used. One-way analysis of variance was used to compare more than three groups. All data were analyzed using the SPSS version 22.0 statistical software, and *P* < 0.05 was considered a significant difference.

## Results

### Brain injury

**Figure 1:**
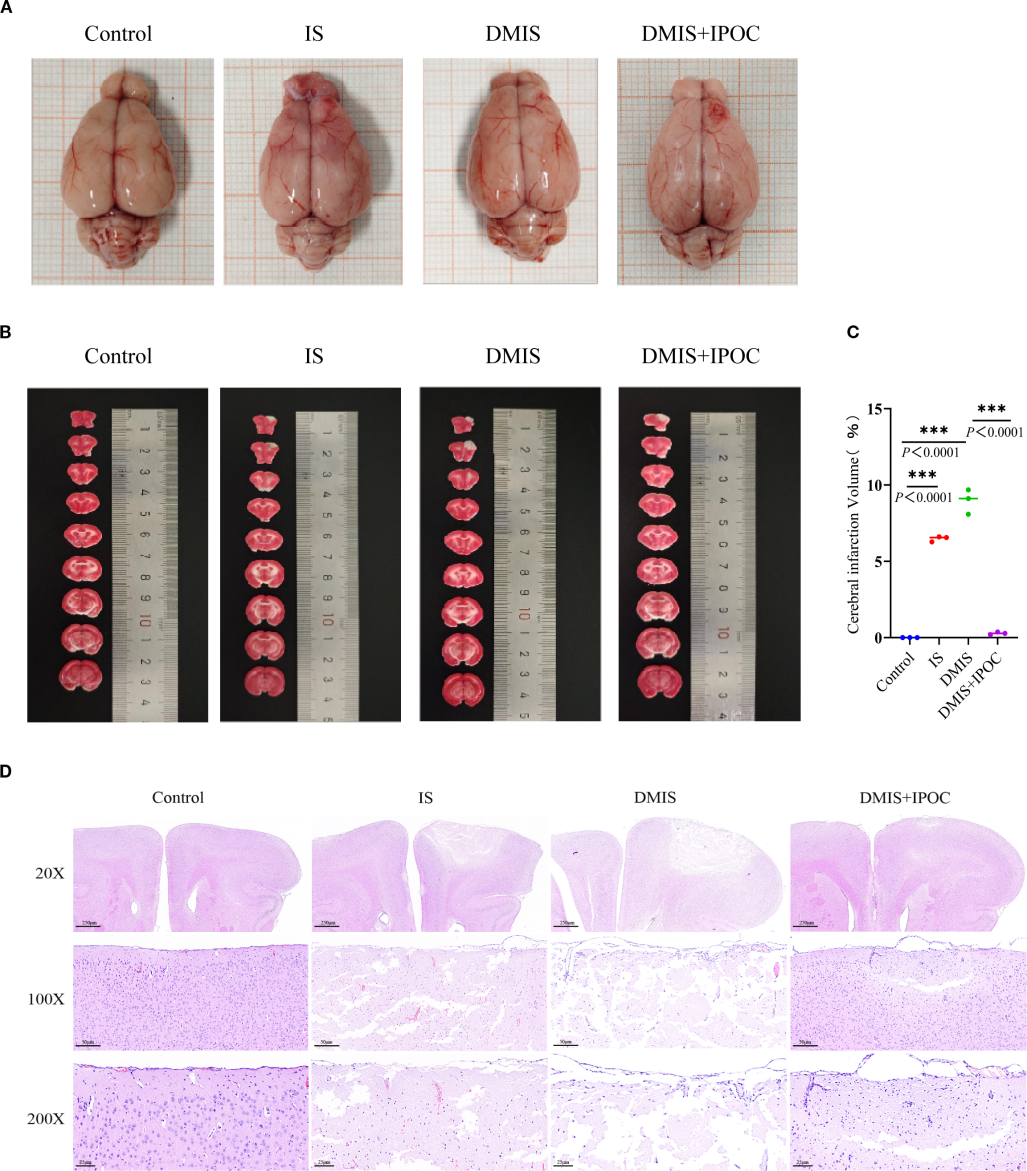
Ischemic postconditioning protects against brain injury in tree shrews with diabetes combined with cerebral ischemia. **(A)** Gross views of brain tissues from each group, showing overall infarct distribution; **(B)** 2,3,5-triphenyltetrazolium chloride (TTC)-stained coronal sections; pale/gray regions indicate infarcted areas, and dark red regions indicate normal viable tissue; **(C)** Quantitative analysis of infarct volume (n = 3); **(D)** Hematoxylin and eosin staining showing pathological changes in the ischemic penumbra. *** indicated *P* < 0.001.

To clarify the neuroprotective effect of IPOC on brain injury under hyperglycemic conditions, this study implemented an IPOC intervention in a tree shrew model of diabetes combined with cerebral ischemia and conducted a pathological assessment of the brain tissue 72 h after the intervention. Quantitative analysis by TTC staining showed that the cerebral infarction volumes in the IS and DMIS groups were higher than those in the control group (*P* < 0.05), whereas the infarction volume in the DMIS + IPOC group was significantly lower than that in the DMIS group (*P* < 0.05) (**Figures 1A–C**). Pathological observations by H&E staining revealed that in the control group, the cortical neurons were arranged in a tightly layered pattern, and the cell morphology was intact (the cell body was round, nuclear membrane clear, and cytoplasm uniform). The IS group presented with typical characteristics of ischemic injury, including neuronal swelling, nuclear pyknosis, axonal rupture, and interstitial edema. Pathological changes in the DMIS group were significantly aggravated, with large areas of necrotic foci, vacuolar degeneration of neurons, and increased inflammatory cell infiltration. In contrast, the DMIS + IPOC group showed a lower degree of injury, lower neuronal necrosis rate, lower density of inflammatory cell infiltration, and better degree of disordered cell arrangement than the DMIS group (**Figure 1D**). These results suggest that both simple cerebral ischemia and cerebral ischemia complicated by diabetes can damage brain nerve cells and cause inflammatory infiltration. However, timely IPOC intervention after ischemia can effectively reduce nerve cell damage and inflammatory infiltration caused by ischemia.

### Neuroinflammation

**Figure 2:**
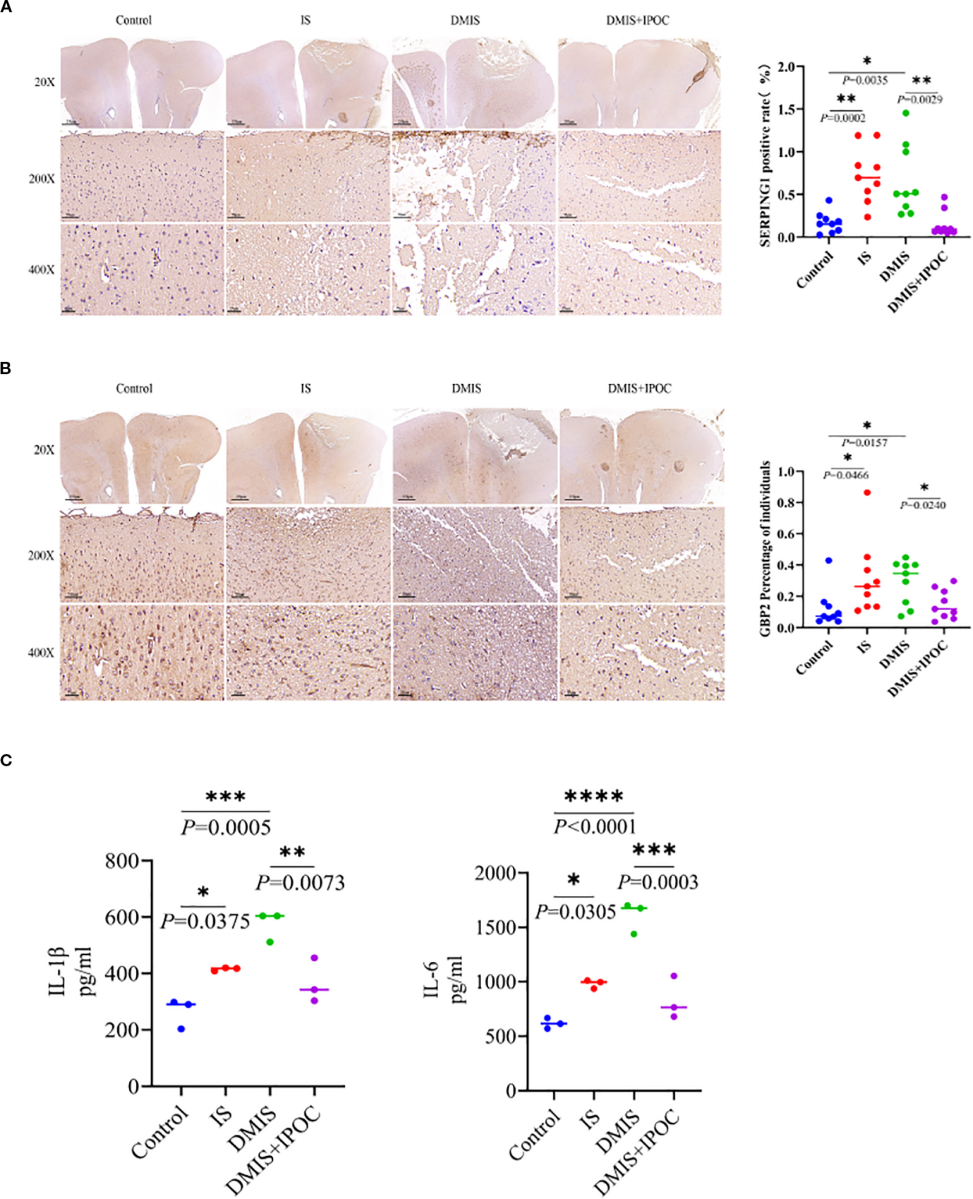
Ischemic postconditioning inhibits the activation of A1 astrocytes and the inflammatory response. **(A)** Immunohistochemistry (IHC) was used to detect the expression level of serping1, a specific marker of A1 astrocytes, in the brain tissue of the ischemic penumbra. **(B)** IHC was USED to detect the expression level of guanine-binding protein 2, an activation marker of A1 astrocytes, in the brain tissue of the ischemic penumbra. **(C)** Enzyme-linked immunosorbent assay was used to detect the expression levels of inflammatory factors interleukin (IL)-1β and IL-6 in the brain tissue of the ischemic penumbra. * indicated *P* < 0.05; ** indicated *P* < 0.01; *** indicated *P* < 0.001; **** indicated *P* < 0.0001.

To further investigate the protective effect of IPOC, the inflammatory response in the ischemic penumbra of the brain was evaluated by detecting the activation of A1 astrocytes and the expression of inflammatory factors in the brain tissue of the ischemic penumbra. IF was used to detect the expression of serping1, a specific marker of A1 astrocytes, and guanine-binding protein 2 (GBP2), an activation marker of A1 astrocytes, in the ischemic penumbra of brain tissues in each group. The results showed that, compared with the control group, the expression levels of serping1 and GBP2 in the IS and DMIS groups were significantly higher (*P* < 0.05), indicating that A1 astrocytes were activated in the IS and DMIS groups. Compared with the DMIS group, the expression levels of serping1 and GBP2 in the DMIS + IPOC group were significantly lower (*P* < 0.05), suggesting that the IPOC intervention after ischemia can effectively inhibit the activation of A1 astrocytes (**Figures 2A, B**). ELISA was used to detect the expression levels of the inflammatory factors IL-1β and IL-6 in the brain tissue of the ischemic penumbra. The results showed that compared with the control group, the levels of inflammatory factors IL-1β and IL-6 in the IS and DMIS groups were significantly higher (*P* < 0.001), indicating an increase in neuroinflammation. Compared with the DMIS group, the levels of the inflammatory factors IL-1β and IL-6 in the DMIS + IPOC group were significantly lower (*P* < 0.01) (**Figure 2C**), suggesting that the IPOC intervention after ischemia can effectively inhibit the neuroinflammatory response after simple and diabetic cerebral ischemia. These results indicate that both simple and diabetic cerebral ischemia may promote cerebral neuroinflammation by activating A1 astrocytes and releasing inflammatory factors. However, timely IPOC intervention after ischemia can effectively inhibit the activation of A1 astrocytes, reduce the release of inflammatory factors, and improve neuroinflammation.

### Mitochondrial autophagy of the microglia

**Figure 3:**
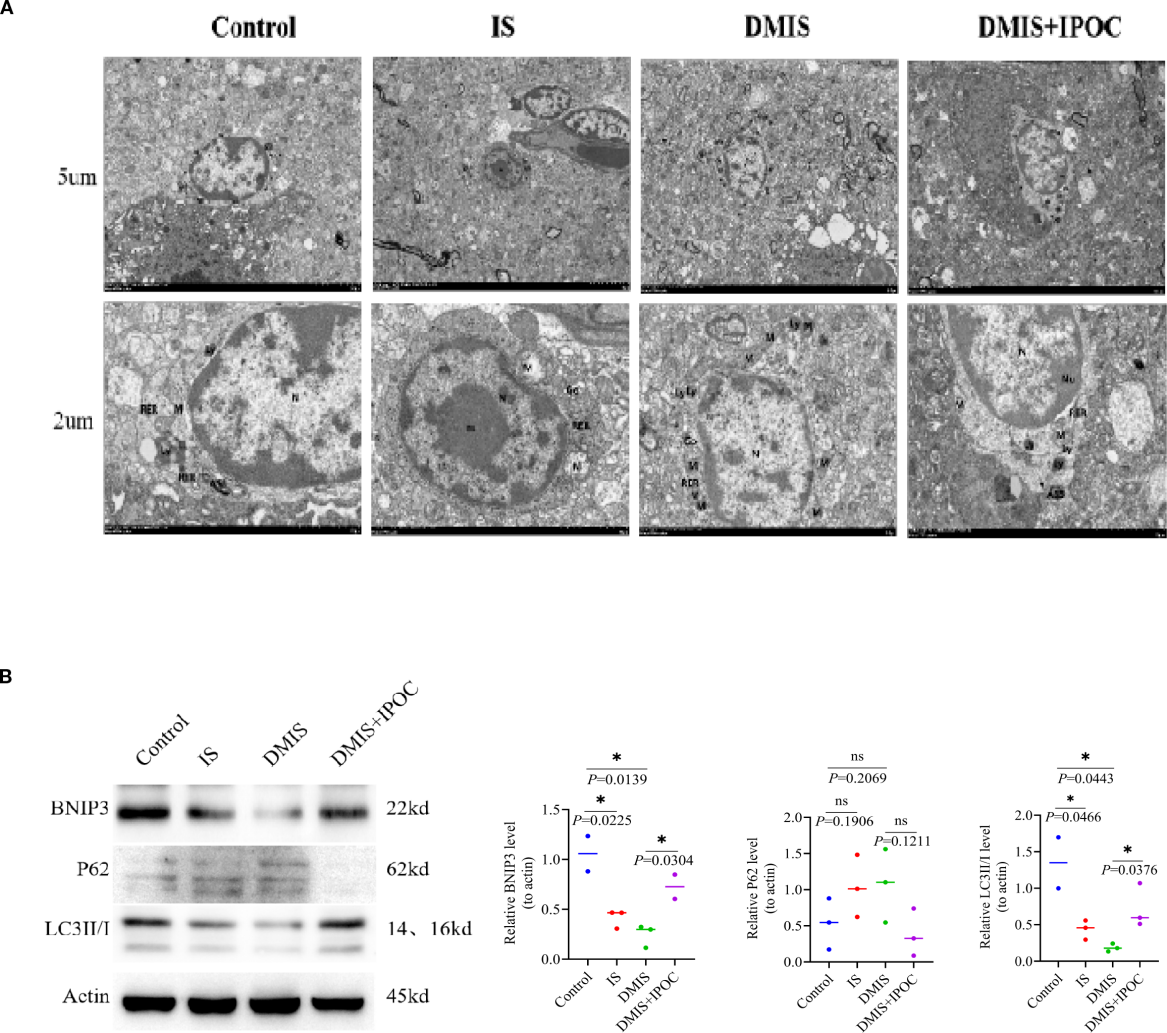
Ischemic postconditioning promotes mitochondrial autophagy in the microglia. **(A)** Transmission electron microscopy was used to detect mitochondrial autophagy in the microglia of the brain tissue in the ischemic penumbra. Note: nucleus (N), double nucleoli (nu), mitochondria (M), rough endoplasmic reticulum, lysosome (Ly), autophagolysosome, and Golgi apparatus (Go). **(B)** Western blotting was used to detect the protein expression levels of LC3 II/I, p62, and Bcl-2/adenovirus E1B 19-kDa-interacting protein 3 in the ischemic penumbra of the brain. * indicated *P* < 0.05. ns, no significant difference.

To investigate the effect of IPOC on mitochondrial autophagy in microglia in the brain tissue of the ischemic penumbra under hyperglycemic conditions, the ultrastructure of the brain tissue in the ischemic penumbra and mitochondrial autophagy in microglia were observed using TEM. The results showed that compared with the control group, the damage to the mitochondria in the microglia in the IS and DMIS groups was significantly higher, whereas mitochondrial autophagy significantly decreased, indicating that mitochondrial autophagy was inhibited in the IS and DMIS groups, leading to an increase in mitochondrial fragments. Compared with the DMIS group, mitochondrial autophagy in the DMIS + IPOC group was significantly higher, suggesting that IPOC intervention after ischemia can effectively promote mitochondrial autophagy and the timely removal of damaged mitochondria (**Figure 3A**).

To further clarify the effect of IPOC on mitochondrial autophagy, the expression of mitochondrial autophagy-related proteins LC3 II/I, p62, and BNIP3 was detected using western blotting (WB). The results showed that compared with the control group, the expression of LC3 II/I and BNIP3 in the IS group was lower, whereas the expression of p62 was higher. However, owing to the large within-group variation, no statistically significant differences were observed. Compared with the control group, the expression of LC3 II/I and BNIP3 in the DMIS group was significantly lower (*P* > 0.05), and the expression of p62 was higher, indicating a reduction in mitochondrial autophagy. Compared with the DMIS group, the expression of LC3 II/I and BNIP3 in the DMIS + IPOC group was significantly higher (*P* < 0.05), and the expression of p62 showed a decreasing trend, suggesting that IPOC can promote mitochondrial autophagy (**Figure 3B**).

### Brain-derived neurotrophic factor–tropomyosin receptor kinase B–hypoxia-inducible factor 1α–Bcl-2/adenovirus E1B 19-Kda-interacting protein 3 axis

**Figure 4:**
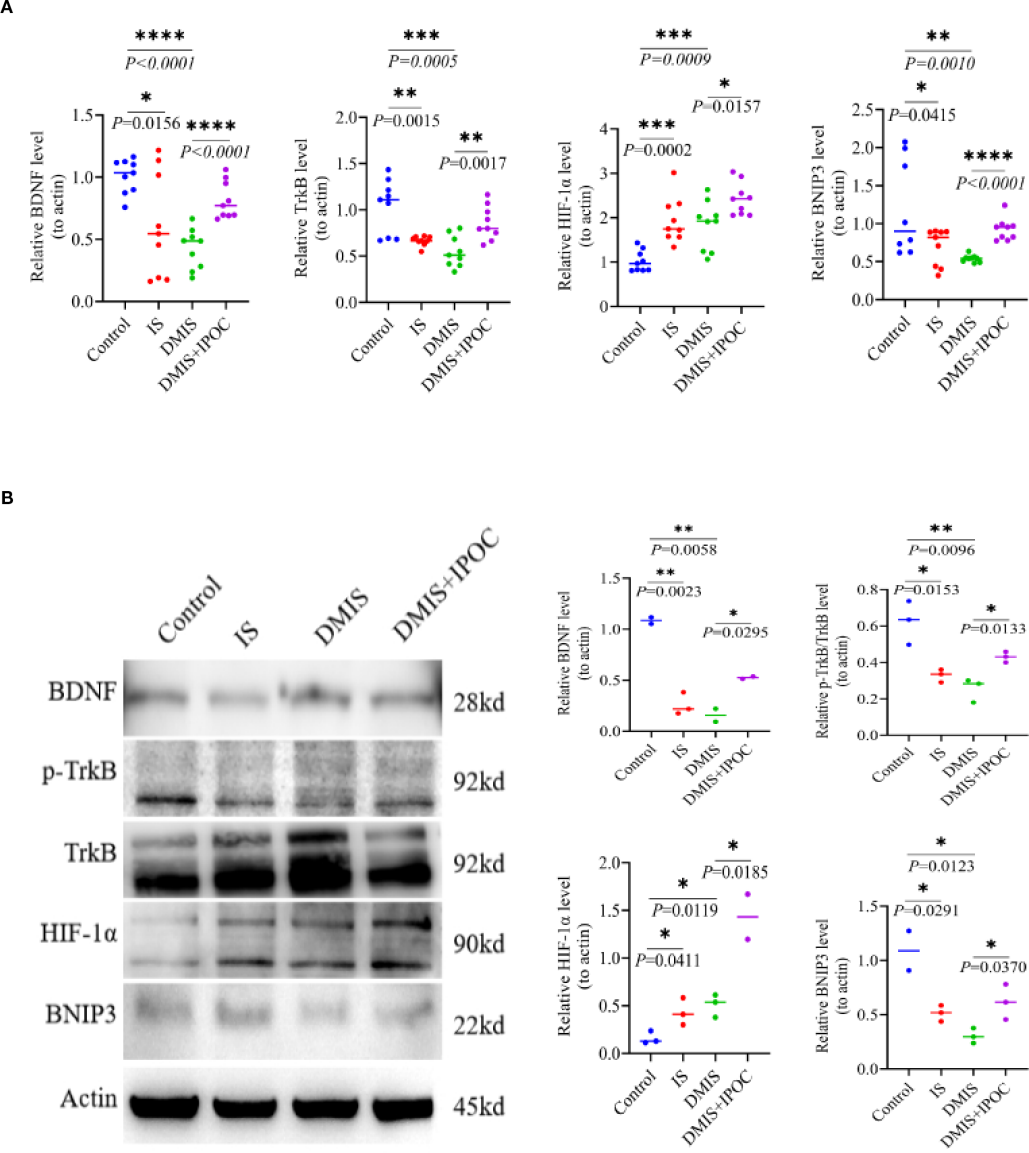
Ischemic postconditioning activates the brain-derived neurotrophic factor/tropomyosin receptor kinase B/hypoxia-inducible factor 1α/Bcl-2/adenovirus E1B 19-kDa-interacting protein 3 (BDNF/TrkB/HIF-1α/BNIP3) axis to promote mitochondrial autophagy. **(A)** Reverse transcription polymerase chain reaction detection shows the mRNA expression levels of BDNF, TrkB, HIF-1α, and BNIP3 in the ischemic penumbra of the brain. **(B)** Western blotting detection shows the protein expression levels of BDNF, TrkB, HIF-1α, and BNIP3 in the ischemic penumbra of the brain. * indicated *P* < 0.05; ** indicated *P* < 0.01; *** indicated *P* < 0.001; **** indicated *P* < 0.0001.

To investigate the possible mechanism by which IPOC activates mitochondrial autophagy in microglia of the brain tissue in the ischemic penumbra under hyperglycemic conditions, the expression of the BDNF/TrkB/HIF-1α/BNIP3 axis was detected using RT-qPCR and WB. The results of RT-qPCR showed that compared with the control group, the mRNA expression levels of BDNF, TrkB, and BNIP3 in the IS and DMIS groups were significantly lower (*P* < 0.05), whereas the mRNA expression level of HIF-1α was significantly higher (*P* < 0.001). Compared to the DMIS group, the mRNA expression levels of BDNF, TrkB, HIF-1α, and BNIP3 in the DMIS + IPOC group were significantly higher (*P*< 0.05) (**Figure 4A**). WB analysis showed that compared with the control group, the total protein expression of BDNF, TrkB, and BNIP3 in the IS and DMIS groups was significantly lower (*P* < 0.05), and the protein expression of HIF-1α was significantly higher (*P* < 0.001). These results suggest that IPOC intervention activates the BDNF/TrkB pathway, upregulates HIF-1α, promotes the expression of BNIP3, and therefore facilitates mitochondrial autophagy (**Figure 4B**).

## Discussion

In this study, we established a pathological model of diabetes combined with cerebral ischemia and systematically elucidated the neuroprotective effects of IPOC on brain injury in hyperglycemic conditions. Both the IS and DMIS groups exhibited remarkable pathological characteristics. The cerebral infarction volume was higher in the IS group than in the control group. Under the synergistic effect of chronic hyperglycemia, the infarct volume in the DMIS group further increased. It is also accompanied by an increase in the blood–brain barrier permeability and upregulation of the levels of pro-inflammatory factors. IPOC intervention significantly reversed the aforementioned injury process, reducing the infarct volume in the DMIS + IPOC group compared to that in the DMIS group. This suggests that its neuroprotective effect may be related to the promotion of mitochondrial autophagy via the BDNF/TrkB/HIF-1α/BNIP3 axis.

TTC and H&E staining systematically revealed the pathological characteristics of cerebral ischemia. (1) The volumes of cerebral infarction in the IS and DMIS groups significantly increased, which is consistent with the clinical feature that diabetes exacerbates ischemic injury (21–23). (2) Histopathological evaluation confirmed that the neuronal necrosis index in the DMIS group was significantly higher than that in the IS group, accompanied by the disruption of the integrity of the blood–brain barrier and the activation of microglia, which verified the vicious cycle theory of “energy metabolism imbalance–excitotoxicity–neuroinflammation” (24). IPOC intervention reduced infarct volume in the DMIS + IPOC group and significantly improved neuronal survival rate. The neuroprotective effect of IPOC may be achieved through a dual regulatory pathway: (1) by activating the PI3K/Akt/mTOR signaling axis, which promotes angiogenesis in the ischemic penumbra (25). (2) It inhibits the activation of the NOD-like receptor family pyrin domain containing 3 inflammasome and imbalances mitochondrial autophagy, thus disrupting the pathological positive feedback of the “oxidative stress–inflammatory storm” (26). This multitarget regulatory characteristic suggests that IPOC may exert a protective effect by reconstructing the homeostasis of the neurovascular unit.

Abnormal activation of A1 reactive astrocytes is a key driving factor (27). This study found that the expressions of serping1 and GBP2, which are markers of A1 astrocytes, were upregulated in both the IS and DMIS groups, accompanied by a significant increase in the levels of pro-inflammatory factors IL-1β and IL-6. This neuroinflammatory storm exacerbates damage through a dual pathway: (1) the release of complement component C1q induces synaptic phagocytosis of neurons and (2) it disrupts the polar distribution of AQP4 in the end feet of astrocytes, leading to leakage of the blood–brain barrier and the formation of an interactive damage network of “glial cells–endothelial cells–neurons” (28). IPOC intervention effectively inhibited A1 astrocyte activation and reduced the levels of inflammatory factors. This indicates that IPOC disrupts the vicious cycle of neuroinflammation. Its potential mechanism of action may be related to the regulation of intracellular signaling pathways in astrocytes. IPOC may inhibit the activation of pro-inflammatory signaling pathways, such as nuclear factor kappa-light-chain-enhancer of activated B cells (NF-κB), which promotes the transformation of astrocytes into the A1 phenotype (29). By suppressing the activation of A1 astrocytes, IPOC reduces the release of neurotoxic substances and inflammator**y** mediators, thereby protecting neurons from injury.

Mitochondrial quality control is a crucial compensatory factor in ischemic stress. Mitophagy maintains cellular homeostasis by mediating the clearance of damaged mitochondria via the PTEN-induced kinase 1/Parkin pathway (30). Ultrastructural analysis showed that both the IS and DMIS groups exhibited inhibited mitophagy in microglia, which manifested as an increase in mitochondrial damage, a decrease in the expression of mitophagy-related proteins LC3 II/I and BNIP3, and an increase in the expression of p62. Impaired mitophagy leads to the accumulation of damaged mitochondria, which in turn generates excessive reactive oxygen species (ROS) and exacerbates cell damage. In contrast, IPOC treatment significantly promoted mitophagy in microglia. In the DMIS + IPOC group, enhanced autophagic flux was observed by TEM; the upregulated expression of LC3 II/I and BNIP3 and the decreasing trend of p62 expression demonstrated an increase in mitophagy (31). The mitophagy-promoting effect of IPOC can effectively remove damaged mitochondria, reduce ROS production, and maintain intracellular homeostasis, thus contributing to the protection of microglia and surrounding neurons.

The results of RT-qPCR and WB indicated that IPOC intervention activated the BDNF/TrkB pathway, upregulated HIF-1α, and promoted the expression of BNIP3. The BDNF/TrkB signaling pathway plays a crucial role in neuronal survival, differentiation, and synaptic plasticity (32). In the context of ischemic brain injury, activation of BDNF/TrkB can promote neuronal repair and protect neurons from apoptosis (33). HIF-1α is a key transcription factor that responds to hypoxia. Under ischemic conditions, the upregulation of HIF-1α can induce the expression of a series of genes involved in angiogenesis, glycolysis, and cell survival, including BNIP3 (34). BNIP3 promotes autophagy and facilitates mitochondrial autophagy (35). Our findings suggest that IPOC activates the BDNF/TrkB pathway, which upregulates HIF-1α. The increase in HIF-1α subsequently promotes the expression of BNIP3, ultimately leading to the activation of mitochondrial autophagy in microglia. Activation of this BDNF/TrkB/HIF-1α/BNIP3 axis may be one of the important mechanisms underlying the protective effect of IPOC in diabetic cerebral ischemia.

Notably, HIF-1α-mediated metabolic adaptation extends beyond transcriptional regulation of BNIP3, encompassing modulation of the pyruvate dehydrogenase kinase (PDK)/pyruvate dehydrogenase complex (PDC) axis—a central node in glucose metabolism (36). Under hypoxic conditions, HIF-1α upregulates PDK expression, which in turn phosphorylates and inhibits PDC, the rate-limiting enzyme that converts pyruvate to acetyl-CoA for TCA cycle entry. This inhibition shifts energy metabolism toward glycolysis, a hypoxia-tolerant pathway that maintains ATP production with reduced mitochondrial oxygen consumption (37). By limiting pyruvate flux into mitochondria, PDK/PDC modulation may reduce excessive ROS generation from electron transport chain dysfunction— a critical trigger for proinflammatory cytokine release (e.g., IL-1β, IL-6) and A1 astrocyte activation (38). Conversely, PDC reactivation restores mitochondrial function, enhances acetyl-CoA and NADH production, and suppresses neuroinflammation in ischemic models (39), suggesting a synergistic role with the BDNF-TrkB-HIF-1α-BNIP3 pathway in maintaining metabolic-immunological homeostasis. In our study, IPOC-induced HIF-1α upregulation may therefore coordinate both mitophagy (via BNIP3) and metabolic reprogramming (via PDK/PDC) to limit free radical-driven injury and promote ATP synthesis, collectively contributing to neuroprotection. Future studies assessing PDK activity, PDC phosphorylation, and TCA cycle intermediates will help dissect this interplay.

A limitation of this study is that we focused on total BDNF expression and did not specifically quantify mature BDNF, the proteolytically processed form that mediates TrkB activation. BDNF is synthesized as a precursor (proBDNF) and cleaved into mature BDNF, which exerts neuroprotective effects via TrkB, while proBDNF may have opposing roles through p75NTR signaling (40). Thus, total BDNF levels may not fully reflect the activity of the mature form. Future studies should quantify mature BDNF specifically, alongside proBDNF, to better correlate BDNF processing with TrkB activation and neuroprotective outcomes in IPOC-mediated effects.

## Conclusion

Our study provides comprehensive evidence that IPOC protects against diabetic cerebral ischemia by inhibiting neuroinflammation and promoting mitochondrial autophagy. The activation of the BDNF/TrkB/HIF-1α/BNIP3 axis may be a key molecular mechanism. These findings not only enhance our understanding of the protective effect of IPOC but also provide potential therapeutic targets for treating diabetic cerebral ischemia. Future studies are required to explore the detailed molecular mechanisms underlying these effects and translate these findings into clinical applications.

## Data Availability

The raw data supporting the conclusions of this article will be made available by the authors, without undue reservation.
