# Supplementary figures and images for: Ischemic postconditioning ameliorates diabetic cerebral ischemia via activating the brain-derived neurotrophic factor–tropomyosin receptor kinase B–hypoxia-inducible factor 1α–Bcl-2/adenovirus E1B 19-kDa-interacting protein 3 pathway to induce microglial mitophagy and suppress A1 astrocyte-mediated neuroinflammation
Source: Front Endocrinol (Lausanne). 2025 Sep 22;16:1620004. doi: 10.3389/fendo.2025.1620004 (PMC12497586; doi:10.3389/fendo.2025.1620004)

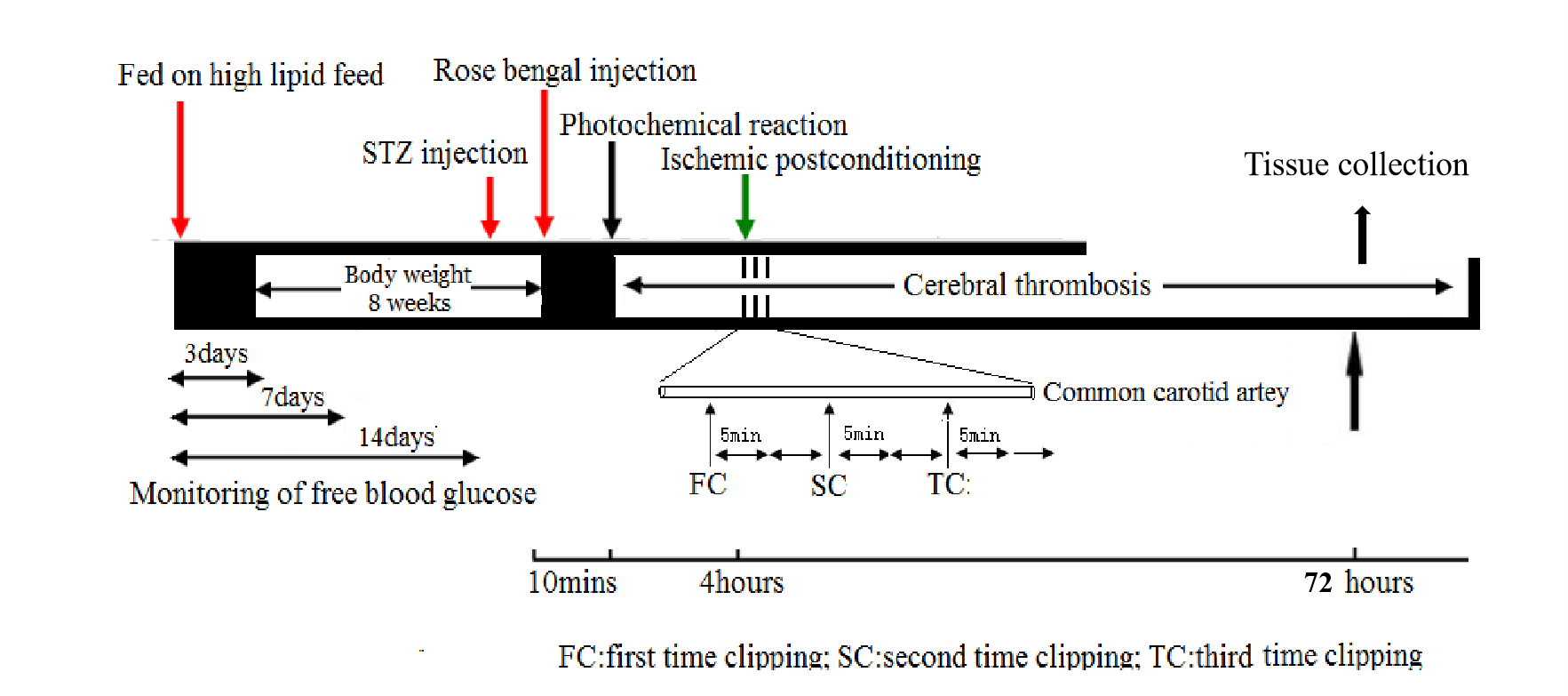

Supplement: Supplementary file 1 [file Image1.tif]

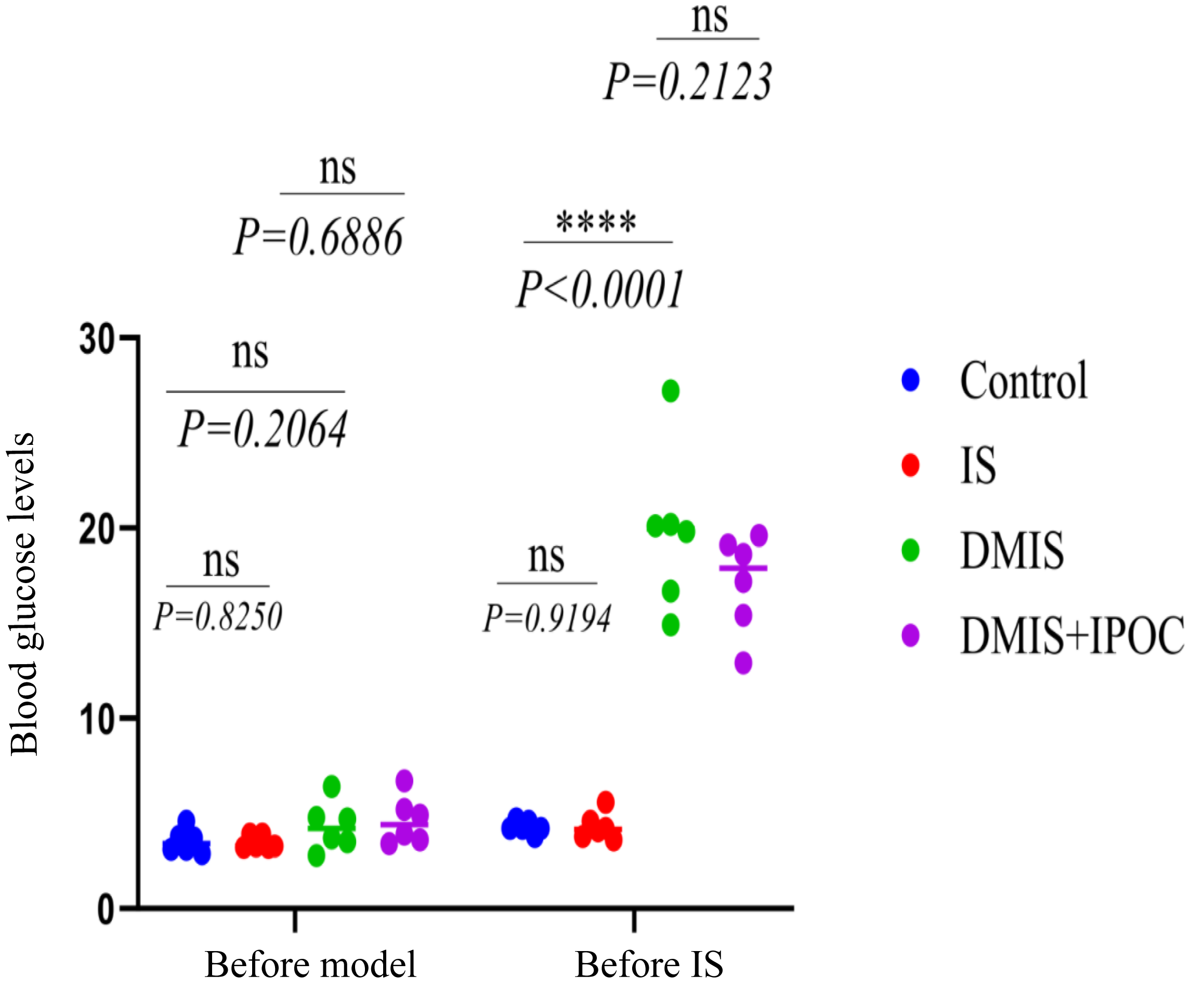

Supplement: Supplementary file 2 [file Image2.tif]
